# Prokinetic Activity of *Prunus persica* (L.) Batsch Flowers Extract and Its Possible Mechanism of Action in Rats

**DOI:** 10.1155/2015/569853

**Published:** 2015-03-02

**Authors:** Wei Han, Jing Dong Xu, Feng Xian Wei, Yong Dong Zheng, Jian Zhong Ma, Xiao Dong Xu, Zhen Gang Wei, Wen Wang, You Cheng Zhang

**Affiliations:** ^1^Department of General Surgery, Hepato-Biliary-Pancreatic Institute, Lanzhou University Second Hospital, Lanzhou 730030, China; ^2^Department of Physiology, School of Basic Medical Sciences, Capital Medical University, Beijing 100069, China; ^3^Disease Control and Prevention Center of Qingan, Tianshui 741600, China; ^4^Department of Pharmacology, Xuanwu Hospital of Capital Medical University, Beijing 100053, China

## Abstract

The peach tree, *Prunus persica* (L.) Batsch, is widely cultivated in China, and its flowers have been used for centuries in traditional Chinese medicine to treat gut motility disorders. But few studies have explored the pharmacological effect of *Prunus persica* (L.) Batsch flowers on gastrointestinal motility. In this study, the activities of different extracts from *Prunus persica* (L.) Batsch flowers on the smooth muscle contractions were evaluated using isolated colon model, and the ethyl acetate extract (EAE) showed the strongest effects in vitro. EAE (10^−8^–10^−5^ g/mL) caused a concentration-dependent stimulatory effect in rat colonic tissue. Additionally, ketotifen (100 *µ*M), cimetidine (10 *µ*M), and pyrilamine (1 *µ*M) produced a significant inhibition of contractions caused by EAE. Furthermore, immunofluorescence and toluidine blue staining revealed increased numbers of mast cells in the EAE group, and EAE increased histamine release from the colonic tissues. These data indicate that EAE has significant prokinetic activity and acts by a mechanism that mainly involves mast cell degranulation. Our study provides a pharmacological basis for the use of an extract of *Prunus persica* (L.) Batsch flowers in the treatment of gut motility disorders.

## 1. Introduction

Gastrointestinal diseases are common public health issues worldwide and gut motility disorders, including indigestion and constipation, are considered to be major causes of ill-health [1]. Although prokinetics and laxatives are used for gut motility disorders, these become less efficient with long-term treatment [2]. Recently, medicinal plants have attracted attention for the treatment of functional dyspepsia and constipation [3, 4] since there is growing evidence that the different components found in medicinal plants have the potential to act synergistically [5]. Medicinal plants are considered to be relatively effective and safe for prolonged treatment, especially in patients with chronic gut motility disorders.

The peach tree,* Prunus persica* (L.) Batsch, which belongs to the family Rosaceae and the genus* Amygdalus* L., is widely cultivated in China and has been commonly used for centuries to treat different diseases. It has been reported that the seeds have anti-inflammatory [6] and antitumor activity [7] and that the nucleus in the seeds can improve blood circulation [8].* Prunus persica* (L.) Batsch leaf extracts showed antihyperglycemic effects on postprandial blood glucose levels in glucose-loaded mice [9] and the major active ingredient was identified as multiflorin A [10]. Several studies have revealed that extracts of* Prunus persica* (L.) Batsch flowers can reduce ultraviolet-induced skin damage and may be useful for protecting against ultraviolet-induced DNA damage and carcinogenesis [11, 12]. In traditional Chinese folk medicine,* Prunus persica* (L.) Batsch flowers have been used as a purgative, or diuretic, and have also been used in cosmetology. Despite a long history of the use of* Prunus persica* (L.) Batsch flowers in traditional Chinese medicine, few studies have explored the pharmacological effect of these preparations on gastrointestinal motility or the underlying mechanism of action.

Thus, the present study aims to investigate the activities of different extracts from* Prunus persica* (L.) Batsch flowers on spontaneous smooth muscle contractions in isolated rat colonic tissue. An additional objective was to explore the mechanisms by which* Prunus persica* (L.) Batsch flowers mediate regulation of gastrointestinal motility.

## 2. Materials and Methods

### 2.1. Plant Material

*Prunus persica* (L.) Batsch flowers were collected in April 2012 from Tianshui city in Gansu province. The plant was identified and authenticated by Professor Sun Xuegang, Institute of Botany, School of Forestry, Gansu Agricultural University. The voucher specimens were retained for future reference at Lanzhou University Second Hospital, China.

### 2.2. Extraction

Dried* Prunus persica* (L.) Batsch flowers (200 g) were extracted with 95% ethanol for 2 h. The extract was then filtered and the filtrate was concentrated using a rotary evaporator. The concentrated residue was extracted sequentially with petroleum ether, chloroform, ethyl acetate, and n-butanol, the residue from each extraction step being used to obtain the subsequent fraction. The extract from each step was evaporated to dryness separately and redissolved in distilled water before use. Four separate extracts were thus obtained: a petroleum ether extract (PEE), a chloroform extract (CE), an ethyl acetate extract (EAE), and an n-butanol extract (NBE).

### 2.3. Drugs and Chemicals

Tetrodotoxin (TTX), indomethacin, atropine, cimetidine, pyrilamine, and ketotifen were purchased from Sigma-Aldrich (Sigma-Aldrich, St. Louis, MO, USA). Petroleum ether, chloroform, ethyl acetate, and n-butanol were purchased from Beijing Chemical Works (Beijing, China). The Krebs solution had the following composition (mM): NaCl, 117; KCl, 4.5; CaCl_2_, 2.5; MgCl_2_, 1.2; NaHCO_3_, 24.8; KH_2_PO_4_, 1.2; glucose, 11.1; pH 7.4.

### 2.4. Animals

Adult male Sprague-Dawley rats (200–250 g) were provided by the Experimental Animal Center of the Xuanwu Hospital, Capital Medical University (Beijing, China). The animals were housed five per cage under normal laboratory conditions (24 ± 1°C) with a controlled 24 h light-dark cycle and were allowed access to standard rodent chow and tap water ad libitum. All experiments were approved by the Ethical Committee on Animal Care of the Lanzhou University Second Hospital and conformed to National Institutes of Health guidelines.

### 2.5. Isolated Tissue Experiments

Following a previously described method [13], isolated distal colon was obtained from adult male Sprague-Dawley rats after cervical dislocation. The abdomen of each animal was opened along the midline. The distal colon was removed and placed in ice-cold oxygenated Krebs solution. The distal colon was then cut along the mesenteric border into the longitudinal muscle fibers and strips of the longitudinal muscle (LM) layer (2 × 8 mm) were prepared. The muscle strips were bathed with Krebs solution (5 mL) recirculating from a chamber maintained at 37 ± 0.5°C during the experiments. The solution was continuously provided with 95% O_2_ and 5% CO_2_. One end of the muscle strip was fixed to the floor of the chamber and the other end was linked to an isotonic transducer (MLT0201, Panlab, Spain) and coupled with a PowerLab (N12128) data acquisition system (AD Instruments; Sydney, Australia) to record motility of the muscle strip computer using chart software LabChart 7 (AD Instruments; Sydney, Australia).

The muscle strips were incubated for 30 min, during which time spontaneous motility of the muscle strips was recorded. After equilibration for 1 h, increasing concentrations of PEE, CE, EAE, and NBE (10^−8^, 10^−7^, 10^−6^, 10^−5^, 10^−4^, and 10^−3^ g/mL) were added to the medium at 10 min intervals and muscle contractions were recorded.

The amplitude and frequency of contractions occurring after administration of each concentration of PEE, CE, EAE, and NBE were determined for 10 min. Relative changes in contractile responses induced by the different concentrations of PEE, CE, EAE, and NBE relative to basal levels (before treatment with those extract) were calculated as percentages.

To investigate the underlying mechanisms of the EAE-induced effects on spontaneous contractions, various blockers or antagonists were added before treatment with EAE. After equilibration for 1 h, atropine (10 *μ*M), TTX (1 *μ*M), norepinephrine (1 *μ*M), indomethacin (10 *μ*M), ketotifen (100 *μ*M), cimetidine (10 *μ*M), and pyrilamine (1 *μ*M) were added to the muscle strips and the spontaneous contractions were measured for 10 min. Increasing concentrations of EAE (10^−8^, 10^−7^, 10^−6^, 10^−5^, 10^−4^, and 10^−3^ g/mL) were then added at 10 min intervals and the muscle contractions were recorded.

### 2.6. Mast Cell Analysis

#### 2.6.1. Staining with Toluidine Blue

Sixteen rats were divided randomly into two equal groups: the control group (*n* = 8) and the experimental group (*n* = 8). Animals in the experimental group were treated with EAE (0.9 g/kg) by gastric administration each day for 7 days based on our preliminary experiment; animals in the control group received the same volume of distilled water at the same dosing intervals. The rats were killed 1 week after administration of the last dose. Colon tissues were removed, fixed in 4% paraformaldehyde in 0.1 M phosphate buffer (pH 7.0), and cut into cross sections with 6 mm thickness. After staining with 0.5% toluidine blue, the tissue sections were examined under light microscopy (100x and 400x), and mast cells were counted using a brightfield microscope. Mast cells were counted in 100 high-power fields in colon preparations of each group. The mast cell density at each site was calculated and recorded as MC numbers mm^−2^.

#### 2.6.2. Immunofluorescence

Rat colon tissues were fixed in 4% paraformaldehyde in 0.1 M phosphate buffer (pH 7.0). Tissues were then cut into cross sections with 10 mm thickness and frozen sections were incubated for 18 h with a mouse monoclonal antibody against tryptase (1 : 100; Abcam Ltd., USA). After washing with PBS, the sections were immersed in Alexa Fluor R488 goat anti-mouse IgG (1 : 300, Invitrogen, USA) for 2 h, then washed with PBS, and mounted in Citifluor. Negative controls were performed by omitting one primary antibody. Tryptase-immunoreactive mast cells were counted using a laser scanning confocal microscope (TCS SP5, Leica, Germany).

#### 2.6.3. Measurement of Histamine Levels

The amount of histamine released by the distal colonic segments in vitro was measured as described previously [14]. Sections of distal colon were equilibrated in Krebs solution with 95% O_2_ and 5% CO_2_ (1 mL) for 30 min; then EAE (10^−6^ g/mL) or the same volume of distilled water was added to the solution. After incubation for 30 min, the supernatant was frozen at −20°C for subsequent analysis of histamine in the supernatant. The tissue segments were dried, weighed, and frozen in liquid nitrogen for subsequent analysis of histamine remaining in the tissue. Histamine levels in both tissue and supernatant were measured using enzyme-linked immunosorbent assay kits (Nanjing Jiancheng Bioengineering Institute, Nanjing, China), used according to the manufacturer's instructions.

### 2.7. Statistical Analysis

Data are expressed as the means ± SEM, with *n* denoting the number of tissue preparations tested. Statistical analyses were performed using one-way analysis of variance followed by Bonferroni correction or Student's *t*-test. *P* value < 0.05 was considered to be statistically significant.

## 3. Results

### 3.1. Effects of* Prunus persica* (L.) Batsch Flowers Extract on the Contractile Responses in Isolated Rat Colon Strips

**Figure 1:**
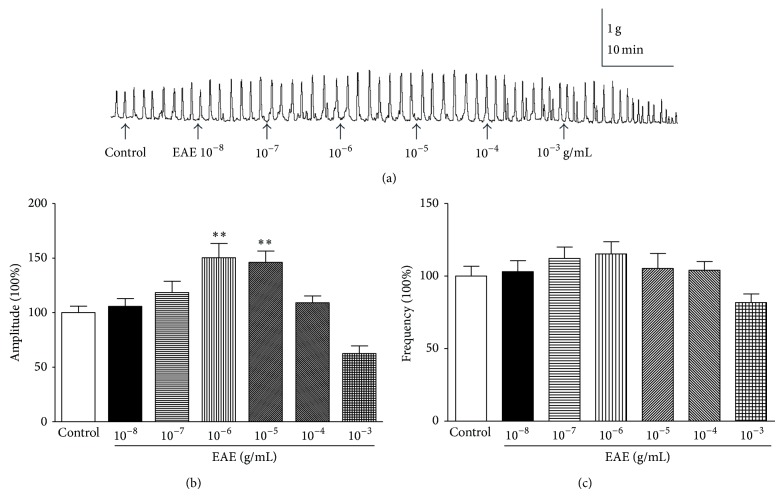
Effects of EAE on the spontaneous contractility of rat colonic muscle strips. (a) Effects of EAE on the spontaneous contractility of rat colonic muscle strips. (b) Group summary data of EAE-induced changes in the amplitude. (c) Group summary data of EAE-induced changes in the frequency. Data are expressed as mean ± SEM (% control, *n* = 6), ^**^ *P* < 0.01, compared to the control groups.

To investigate the effects of PEE, CE, EAE, and NBE, we examined the effects of isolated rat colon muscle motility at doses of 10^−8^, 10^−7^, 10^−6^, 10^−5^, 10^−4^, and 10^−3^ g/mL. Only EAE showed the strongest effects and was used in the subsequent experiments (Figure 1(a)). PEE, CE, and NBE have no effect on isolated rat colon muscle motility (data not shown). EAE concentration dependently increased the frequency and amplitude of colon muscle contractions in the concentration range 10^−8^–10^−5^ g/mL but exerted an inhibitory effect at higher concentrations (Figure 1(a)). It has previously been shown that low concentrations of herbal medicines enhance the contractions of jejunal smooth muscle while high concentrations reduce the contractions [15]. The contractile effect (amplitude 150.4 ± 13.12%, frequency 115.2 ± 8.46%) in the presence of EAE at a concentration of 10^−6^ g/mL was comparable to the basal level (Figures 1(b) and 1(c)). EAE induced a definite contraction of the longitudinal muscle in the isolated rat colon strip at a dose of 10^−6^ g/mL (Figure 1).

### 3.2. Impact of Antagonists on EAE Fraction-Induced Smooth Muscle Contractions

We further investigated the mechanism underlying the action of EAE on muscle contractions. Muscle strips were incubated for 30 min, during which time spontaneous motility of the muscle strips was observed. After equilibration for 1 h, the tissue was treated for 10 min with atropine (10 *μ*M), tetrodotoxin (TTX) (1 *μ*M), norepinephrine (1 *μ*M), indomethacin (10 *μ*M), ketotifen (100 *μ*M), cimetidine (10 *μ*M), and pyrilamine (1 *μ*M), which abolished the contractile effect. Doses of 10^−8^, 10^−7^, 10^−6^, 10^−5^, 10^−4^, and 10^−3^ g/mL of EAE were then added separately; responses to EAE were demonstrated to be concentration-dependent.

**Figure 2:**
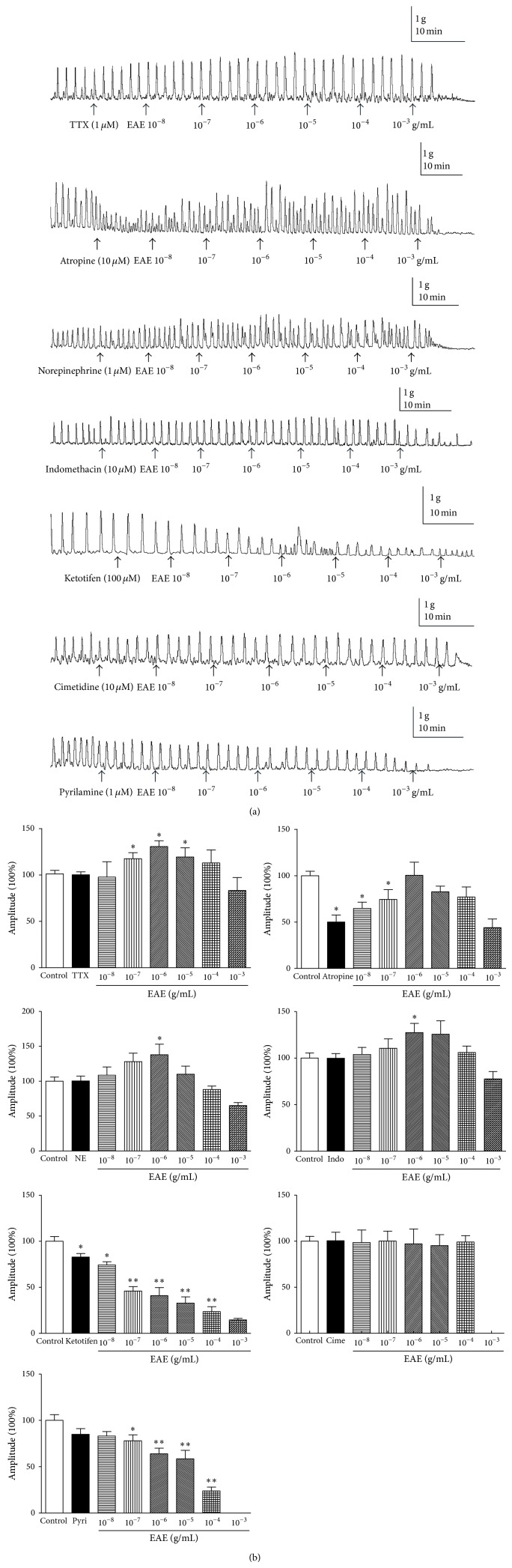
(a) Effects of EAE after 10 min pretreatment of the tissues with TTX (1 *μ*M), atropine (10 *μ*M), norepinephrine (1 *μ*M), indomethacin (10 *μ*M), ketotifen (100 *μ*M), cimetidine (10 *μ*M), and pyrilamine (1 *μ*M). EAE (10^−8^, 10^−7^, 10^−6^, 10^−5^, 10^−4^, and 10^−3^ g/mL) was added to the bath solution. (b) Statistical analysis of EAE on the contractility of rat colonic tissue pretreatment with tetrodotoxin (TTX), atropine, norepinephrine (NE), indomethacin (Indo), ketotifen, cimetidine (Cime), and pyrilamine (Pyri), respectively. The mean contractile amplitude of rat colonic tissue in normal contractile state is set as 100% (control). Data are expressed as mean ± SEM (% control, *n* = 6) ^*^ *P* < 0.05, ^**^ *P* < 0.01, compared to the control groups.

The effect of TTX on the EAE-induced increased response was studied. It has been established that the enteric nervous system can regulate intestinal motility [16] and TTX is a useful pharmacological tool that can distinguish between neuronal and myogenic responses in isolated smooth muscle sections [17, 18]. We therefore used TTX to distinguish whether the EAE-induced increase in frequency of spontaneous contractions of isolated rat colon section was mediated by the enteric nervous system. TTX (1 *μ*M) affected spontaneous contractions of isolated rat colon strip but did not prevent the EAE-induced contraction (Figure 2), indicating that enteric neurons are not involved in the EAE-induced contraction. Atropine, a muscarinic receptor antagonist, was also used to explore the mechanism of the EAE-induced contraction. Pretreatment with atropine (10 *μ*M) did not inhibit the EAE-induced contraction (Figure 2), suggesting that cholinergic secretomotor neurons are also not involved in this process.

It has been reported that adrenergic pathways can modulate gut motility [19]. Norepinephrine (NE), a nonselective adrenergic agonist, was also used to explore the EAE-induced contraction. Pretreatment with NE (1 *μ*M) did not inhibit the EAE-induced contraction (Figure 2), suggesting that adrenergic pathways are not involved in EAE-induced contraction.

Additionally, to determine if the effects of EAE were due to the release of prostaglandins (PGs), we tested the EAE-induced response in the presence of indomethacin, an inhibitor of cyclooxygenase. Indomethacin (10 *μ*M), did not abolish the EAE-induced contraction response (Figure 2) suggesting that the EAE-induced effect on muscle motility might not be involved in PGs.

Mast cells have been reported to be associated with gastrointestinal motility in various conditions [20]. Mast cells produce histamine which can stimulate enteric smooth muscle contraction [21, 22]. Interestingly, pretreatment with ketotifen (100 *μ*M), a mast cell membrane stabilizer, inhibited the EAE-induced contraction (Figure 2). It is known that the major product of mast cell is histamine [23]. Thus, the involvement of histamine in EAE-induced response was investigated by the H_1_ receptor antagonist pyrilamine and the H_2_ receptor antagonist cimetidine. Pretreatment with cimetidine (10 *μ*M) or pyrilamine (1 *μ*M) could inhibit the EAE-induced contraction (Figure 2). Based on those results, it is demonstrated that EAE-induced enteric smooth muscle contraction is largely mediated by mast cell degranulation.

### 3.3. Changes in Mast Cells in the EAE-Treated Group

**Figure 3:**
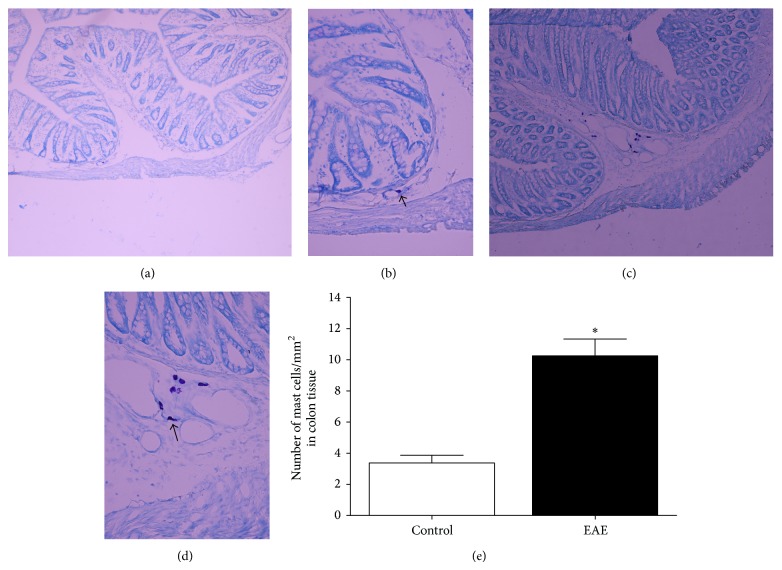
Toluidine blue staining of colonic sections reveals well-defined mast cells predominantly in the EAE group and control group. Mast cells in the EAE-treated group showed an increase in cell volume together with signs of degranulation compared with the control group. Control sample of rat colon ((a) magnification ×100; (b) ×400). EAE-treated sample of rat colon ((c) magnification ×100; (d) ×400). (e) Number of colonic mast cells in control group and the EAE group. Data are expressed as mean ± SEM (*n* = 8) ^*^ *P* < 0.05 compared to the control groups.

To investigate the role of mast cells in gastrointestinal motility induced by EAE, rat colon sections were stained with toluidine blue, and mast cells were identified by their metachromatic staining. The EAE-treated group was found to have many more mast cells in the colon compared with the control group; the number of mast cells/mm^2^ increased from 3.36 ± 1.41 to 10.25 ± 3.06 (*n* = 8, *P* < 0.05, Figure 3). Mast cells in the EAE-treated group also showed an increase in cell volume together with signs of degranulation compared with the control group (Figure 3).

**Figure 4:**
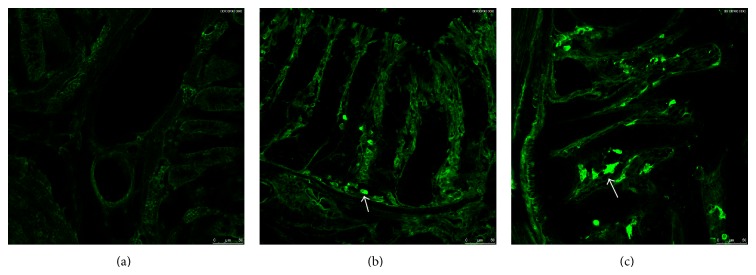
Mast cells were determined in the EAE-treated group and control group. (a) Omission of primary antibody. (b) Detection of tryptase in control group. (c) Detection of tryptase in EAE-treated group.

Mast cells numbers and distribution were then examined by immunofluorescence of colon sections, which can reveal circumscript staining for mast cell tryptase. This technique also showed increased numbers of mast cells in the EAE group compared to the control group (Figure 4). Taken together, these data demonstrate that EAE-induced gastrointestinal motility was highly associated with mast cell activation.

### 3.4. Release of Histamine

**Figure 5:**
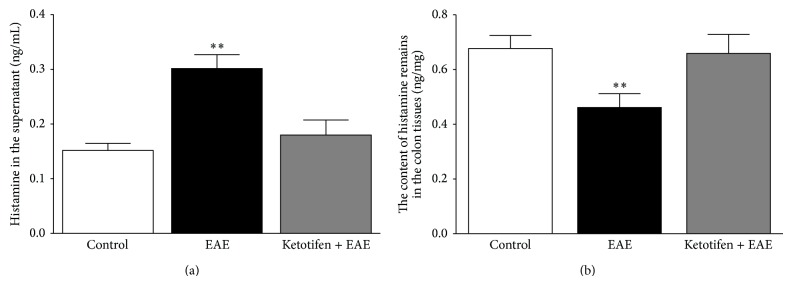
EAE-induced histamine release from the rat colonic tissues. (a) Pretreatment of tissues of rat colon with EAE (10^−6^ g/mL) increased the concentration of histamine in the supernatant; pretreatment of the tissues with ketotifen (100 *μ*M) prevented EAE-induced histamine release into the supernatant. (b) The histamine concentration in colon tissues treated with EAE was decreased; pretreatment of the colon tissues with ketotifen prevented the EAE-induced reduction in tissue histamine. Data are expressed as mean ± SEM (*n* = 8) ^**^ *P* < 0.01 compared to the control groups.

To further explore the effect of histamine release by mast cells involved in EAE-induced gastrointestinal motility, we measured histamine release in rat colonic tissues treated with EAE. The basal concentration of histamine in the supernatant of rat colon tissue was 0.15 ± 0.02 ng/mL (*n* = 8). Following treatment of the colon tissue with EAE, the histamine concentration increased to 0.30 ± 0.03 ng/mL (*P* < 0.05, *n* = 8). If the colon tissues were pretreated with ketotifen (100 *μ*M) for 10 min before adding the EAE, the histamine concentration was 0.18 ± 0.03 ng/mL (*P* > 0.05, *n* = 8), a value close to the basal level (Figure 5(a)).

Simultaneously, the concentration of histamine remaining in the colon tissue was examined. The basal concentration of histamine in colon tissue was 0.68 ± 0.05 ng/mL (*n* = 8). The histamine concentration in colon tissue treated with EAE decreased to 0.46 ± 0.05 ng/mL (*P* < 0.05, *n* = 8). Following pretreatment of the colon tissue with ketotifen (100 *μ*M) for 10 min before adding the EAE, the histamine concentration in the tissue was 0.66 ± 0.07 ng/mL (*P* > 0.05, *n* = 8), suggesting that ketotifen can inhibit EAE-induced histamine release (Figure 5(b)).

## 4. Discussion

*Prunus persica* (L.) Batsch flowers have been used to treat indigestion and constipation in China since ancient times, but the mechanism of action of this herbal remedy is not fully understood. The gastrointestinal activity of EAE in rats was found to be mediated by effects on spontaneous smooth muscle contractions in isolated rat colonic tissue. Mast cell analysis and measurement of histamine levels in rat colon tissues confirmed that the mechanism underlying the gastrointestinal activity involves release of histamine from mast cells.

In the present study, the activities of PEE, CE, EAE, and NBE on the smooth muscle contractions were evaluated using isolated rat colon model; only EAE exhibited a significant biphasic dose-response profile in rat gastrointestinal motility. We have provided evidence that low dose EAE increased gastrointestinal motility, while high doses had inhibitory effects. Biphasic effects of herbal medicine extracts on gastrointestinal motility [24, 25], antihyperglycemic activity [26], and cellular proliferation [27] have previously been demonstrated. Our data suggest that EAE has a significant stimulatory effect on spontaneous smooth muscle contractions in isolated rat colonic tissue. We further investigate the mechanism underlying the action of EAE on muscle contractions.

To explore the mechanisms of the observed prokinetic effects of EAE, we carried out in vitro experiments with rat colonic tissues using methods previously described for studying the gut-stimulatory effects of plant extracts [13]. The effect of antagonists on EAE-induced smooth muscle contraction was also determined. Modulation of gastrointestinal muscular activity is dependent on the interaction of the central nervous system and the enteric nervous system. The enteric nervous system (ENS) regulates vital gut functions such as motility, epithelial transport, microcirculation, and barrier function [28]. It has also been reported that the enteric nervous system influences gastrointestinal motor activity. The effect on the contraction response induced by EAE was not affected by the nerve blocker, TTX, suggesting that the pharmacological effects were not mediated through the nerves. A muscarinic receptor antagonist, atropine, was used to determine whether the gut-stimulatory effect was mediated through cholinergic activity. In the presence of atropine, the contractile effect of EAE was not blocked, indicating that cholinergic pathways are not responsible for the stimulant effect. It has been reported that adrenergic pathways can modulate gut motility [19] but we found that the contractile effect of EAE was not influenced by NE, a nonselective adrenergic agonist, suggesting that the EAE-induced effect is not mediated through adrenergic pathways. PGs have been shown to contract longitudinal smooth muscles [29], and indomethacin, a nonselective cyclooxygenase inhibitor, can relax the longitudinal intestinal smooth muscles [30]. We found that indomethacin did not abolish the EAE-induced contraction response. Histamine and PGs are key chemical messengers released by mast cells, and it has been reported that histamine stimulates the generation of prostaglandin F2*α* from smooth muscle [31]. We therefore also investigated the role of mast cells and histamine in EAE-induced activity.

The regulation of gastrointestinal motility is a complex interplay between the enteric nervous system, gastrointestinal hormones, and immune cells. Mast cells play important roles in both innate and adaptive immunities and are involved in many processes including food allergies, visceral hypersensitivity, gastrointestinal motility, and intestinal cancer [32]. Mast cells, interacting with other cell types, may influence motor and sensory functions in the human gastrointestinal tract [33]. It has also been reported that mast cells are increased in the colonic mucosa of patients with irritable bowel syndrome (IBS) [34], chagasic megacolon [35], and Hirschsprung's disease [36] and are correlated with abdominal pain and diarrhea in these patients [37, 38]. Recently, mast cells have been shown to be increased in constipated patients as a compensatory mechanism for impaired propulsive activity in these patients [38]. We found that pretreatment with the mast cell membrane stabilizer, ketotifen (100 *μ*M), inhibited EAE-induced contraction, suggesting that EAE-induced enteric smooth muscle contraction is largely mediated by mast cell degranulation. It has been reported that histamine H_1_ and H_2_ receptors are involved in gastrointestinal motility [39, 40], and we found that the contractile effect of EAE was abolished by H_1_ receptor antagonist pyrilamine and the H_2_ receptor antagonist cimetidine, suggesting that the EAE-induced effect is mediated through histamine release. Mast cell analysis was then used to confirm the involvement of mast cells in the EAE-treated group. Toluidine blue staining and immunofluorescence showed that, following treatment with EAE, the number of mast cells was increased compared with controls. Following pretreatment of rat colon tissues with EAE, the histamine content in the supernatant was higher than that in the control group. These results provide further evidence that EAE-induced contraction responses are mediated via mast cell degranulation, which increases histamine levels in the intestine.

## 5. Conclusions 

In summary, EAE was shown to stimulate rat colonic muscle contraction in a dose-dependent manner. The numbers of mast cells and the level of histamine were increased in the EAE group and the stimulatory effect of EAE was completely blocked by ketotifen. The data demonstrate that the stimulatory action of EAE on gastrointestinal motility is mediated by mast cell degranulation, which further increases histamine levels in the gut. This study therefore provides a scientific basis for the medicinal use of* Prunus persica* (L.) Batsch in gut motility disorders such as indigestion and constipation. However, more advanced evidence is required before EAE is employed as a potential therapeutic drug in the clinic.
